# Optimization of experimental conditions of microbial desulfurization in coal mine using response surface methodology

**DOI:** 10.3389/fbioe.2022.1076814

**Published:** 2022-11-25

**Authors:** Chun‐ming Ai, Ping‐ping Sun, Dan Zhao, Xiao‐zhi Mu

**Affiliations:** ^1^ College of Safety Science and Engineering, Liaoning Technical University, Huludao, China; ^2^ Key Laboratory of Thermal Disaster and Prevention, Ministry of Education, Huludao, China; ^3^ Shanxi Jinshen Shaping Coal Industry Co, Ltd., Xinzhou Shanxi, China

**Keywords:** microbial desulfurization, response surface methodology, experimental optimization, plackett-burman design method, spontaneous combustion

## Abstract

To reduce the risk of spontaneous combustion during coal storage and transportation, microbial desulfurization technology is used to reduce the content of inorganic sulfur in coal. A strain of *Aciditithiobacillus ferrooxidans* was purified from coal mine water in Datong, Shanxi Province, and its desulfurization test conditions were optimized. Taking the inorganic sulfur removal rate of coal as the response value. The Plackett-Burman design method was used to screen the main factors affecting the response value. And the response surface method was used to establish the continuous variable surface model to determine the interaction between the factors. The results show that the three main factors affecting the response value and their significance order are temperature > coal particle size > desulfurization time, and the interaction between temperature and coal particle size has the greatest effect. When the temperature is 29.50°C, the coal size is 100 mesh, and the desulfurization time is 11.67 days, the desulfurization effect is the best, and the removal rate of inorganic sulfur can reach 79.78%, which is close to the predicted value, and the regression effect is wonderful.

## 1 Introduction

China is rich in coal types, with high sulfur coal reserves accounting for about 1/3 of the total coal, of which 20% of coking coal has sulfur content greater than 2%, 30% of fat coal belongs to high sulfur coal, and 60% of gas coal belongs to high sulfur coal (Qin, 2000; Zhao et al., 2003). During coal storage and transportation, the sulfur (mainly pyrite sulfur) distributed in the coal makes the coal storage pile prone to oxidation and spontaneous combustion. The spontaneous combustion of coal pile not only threatens personnel safety, pollutes the environment, damages equipment, but also causes huge economic losses (Zhang and Xie, 2001; Duan et al., 2015; Qu et al., 2021). To effectively remove sulfur from coal, scholars put forward relevant desulfurization technological measures from the aspects of physics, chemistry and microorganism (Ayhan et al., 2005; Liu et al., 2019; Sun et al., 2021). Microbial desulfurization uses the metabolic activities and metabolites of bacteria to dissolve inorganic sulfide and some organic sulfur in coal and reduce the content of sulfur in coal, which reduces effectively the risk of spontaneous combustion during coal storage and transportation.

Microbial desulfurization technology has the advantages of clean and environmental protection, low process cost, simple process, low energy consumption, mild reaction conditions and so on. Domestic and foreign scholars have carried out a large number of studies on microbial desulfurization (Gao et al., 2014; Xu et al., 2019; Kotelnikov et al., 2020; Zhao et al., 2021). In 1922, Rudolf et al. (Rudolfs, 1922; Rudolfs and Helbronner, 1922) were the first to report bacterial leaching of pyrite and zinc sulfide ore. In 1947, Clomer and Hinkle (Colmer and Hinkle, 1947) were the first to isolate *Aciditithiobacillus ferrooxidans* (*A.f*), which oxidized metal sulfides, from acidic mine water in coal mines. In the 1980s, Kargi, Monticello, Olson et al. (Kargi, 1982; Monticello and Finnerty, 1985; Olson and Brinckman, 1986) began to study the dissolution of pyrite in coal measures by microorganisms on the basis of bio-metallurgy. Tao et al. (Tao et al., 2005; Tao et al., 2007) applied electrochemistry to microbial desulfurization process, which not only promoted the growth of *Tf*, but improved the adsorption between coal and bacteria, and achieved good results. Xu et al. (Xu et al., 1990) used self-screened *Tf* to react with coal for 8 days, and the total sulfur content decreased from 2.45% to 1.12%, in which the pyrite removal rate was 70%. Microbial desulfurization has become the research focus and development trend in the field of coal desulfurization.

To improve the removal rate of inorganic sulfur from coal by bacteria, the response surface analysis method was used to optimize the test conditions. Response surface analysis method has fewer experiments and short cycle, and obtains the regression equation of each factor and response value, which has advantages in the optimization of test conditions (Ai et al., 2016; Ataallahi et al., 2021). Taking the inorganic sulfur removal rate as the response value, firstly, the important factors affecting the inorganic sulfur removal rate were selected by Plackett-Burman (PB) design method. Then, the steepest climbing test was used to approach the maximum corresponding regions. Finally, response surface analysis method was used to establish a continuous variable surface model to optimize the important factors of inorganic sulfur removal rate.

## 2 Materials and principles

### 2.1 Materials

#### 2.1.1 Coal sample

The coal samples used in the test were taken from a coal mine in Datong, Shanxi Province. The coal storage pile of the coal mine had spontaneous combustion accidents. The industrial analysis results of coal samples were shown in Table 1.

**TABLE 1:** Industrial analysis of coal samples.

Project	Total water (%)	Analysis water (%)	Ash content (%)	Fixed carbon
Content	5	0.8	20	65
Project	Volatile matter (%)	Total sulfur (%)	Pyrite sulfur (%)	Calorific value (MJ/kg)
Content	18	2.39	1.35	28

The total sulfur content in the coal sample is 2.39%, which belongs to medium-high sulfur coal, and there is a great risk of spontaneous combustion. The sulfur content of pyrite is 1.35%, accounting for 56.49% of total sulfur. After mechanical crushing, the coal samples were sieved into different particle sizes according to the test requirements and sealed for storage.

#### 2.1.2 Experiment strains

In this experiment, the mine water was used as the raw solution for strain screening. After filtering the collected mine water, 10 ml filtrate was taken into a conical flask filled with 100 ml 9 K solution medium (as shown in Table 2) and put into a 150 r/min, 30°C shaker to observe the color changes. The color of the medium gradually changed from light green to light yellow, and finally showed reddish brown. Then the bacteria solution was inoculated into a new 9 K liquid medium for enrichment and culture. This process was repeated three times.

**TABLE 2:** Composition of 9 K medium.

Serial number	Name	Chemical formula	Suppliers	Mass fraction	Content (g/L)
1	Ammonium sulfate	(NH4)2SO4	Sinopharm Chemical Reagent Co., Ltd.	≥0.990	3.0
2	Potassium chloride	KCl	Sinopharm Chemical Reagent Co., Ltd.	≥0.995	0.1
3	Dipotassium hydrogen phosphate	K2HPO4	Sinopharm Chemical Reagent Co., Ltd.	≥0.990	0.5
4	Magnesium sulfate	MgSO4·7H2O	Sinopharm Chemical Reagent Co., Ltd.	≥0.990	0.5
5	Calcium nitrate	Ca(NO3)2	Sinopharm Chemical Reagent Co., Ltd.	≥0.985	0.01
6	Ferrous sulfate	FeSO4·7H2O	Sinopharm Chemical Reagent Co., Ltd.	≥0.990	44.2

1 ml bacterial solution was taken from the latest culture medium, diluted with sterile distilled water, and then coated in 9 K solid medium. The culture dish was wrapped with sealing film (to avoid miscellaneous bacterial infection) and placed in a constant temperature biochemical incubator at 30°C for constant temperature to culture. The color changes of the medium were observed.

Several colonies of the same size and color were selected when colonies growing on the solid medium, and prepared with 10 ml sterilized deionized water respectively to prepare suspension. A character line of “Z” was marked on 9 K solid medium. After the growth of colonies, well-shaped colonies were selected from the solid medium to prepare suspension.

The above process was repeated until the microbe community with the same shape were observed under the microscope, and the purified bacteria was identified as *A.f*.

### 2.2 Principle

*A.f* are mainly based on electrochemical mechanism in the desulfurization process, and carry out chemoautotrophic growth in the reaction process (Fowler et al., 2001).

Early desulfurization period (a small amount of Fe^2+^ exists in the desulfurization system):

$${4\text{Fe}}^{2+}{+4H}^{+}{+O}_{2}\overset{microorganism}{\to }{4\text{Fe}}^{3+}{+2H}_{2}O$$ (1)

$${\text{FeS}}_{2}{+8H}_{2}{O+14\text{Fe}}^{3+}\to {16H}^{+}{+2\text{SO}}_{4}^{2-}+{15\text{Fe}}^{2+}$$ (2)

Mid desulfurization period (a large amount of Fe^2+^ exists in the desulfurization system):

$${2\text{FeS}}_{2}{+2H}_{2}{O+7O}_{2}\overset{microorganism}{\to }{4H}^{+}{+4\text{SO}}_{4}^{2-}{+2\text{Fe}}^{2+}$$ (3)

$${\text{FeS}}_{2}{+2\text{Fe}}^{3+}\to {3\text{Fe}}^{2+}+2S$$ (4)

Later desulfurization period (a large amount of Fe^3+^ exists in the desulfurization system):

$${2S+2H}_{2}{O+3O}_{2}\overset{microorganism}{\to }{2H}_{2}{\text{SO}}_{4}$$ (5)

$${S+6\text{Fe}}^{3+}{+4H}_{2}O\to {6\text{Fe}}^{2+}{+\text{SO}}_{4}^{2-}+8H^{+}$$ (6)

The nutrient sources in the process of bacterial desulfurization mainly come from iron in 9 K medium and sulfur in coal, so as to remove sulfur from coal.

## 3 PB experiment

### 3.1 Experiment design

According to the early stage of the single factor experiment, the possible factors affecting coal desulfurization reaction were obtained, including coal particle size (A), bacterial solution (B), time (C), coal consumption (D), temperature (E), shaking table speed (F) and initial pH value (G). The Plackett-Burman design with *N* = 11 is selected. To consider the error, four virtual groups were set, two levels were taken for each factor, +1 indicated high level and -1 indicated low level, as shown in Table 3. The significance of each factor was analyzed by Minitab software, and the factors with *p* < 0.01 were selected as the main influencing factors, and the main factors affecting the inorganic sulfur removal rate were obtained.

**TABLE 3:** Plackett-Burman experiment design.

Serial number	Facts											The inorganic sulfur removal rate/%
	A	B	C	D	E	F	G	H	I	J	K	
1	-1	-1	-1	-1	-1	-1	-1	-1	-1	-1	-1	67.04
2	1	-1	-1	-1	1	1	1	-1	1	1	-1	62.70
3	-1	-1	1	1	1	-1	1	1	-1	1	-1	86.31
4	-1	1	1	1	-1	1	1	-1	1	-1	-1	57.22
5	-1	-1	-1	1	1	1	-1	1	1	-1	1	80.25
6	1	-1	1	1	-1	1	-1	-1	-1	1	1	61.57
7	-1	1	1	-1	1	-1	-1	-1	1	1	1	88.92
8	1	1	-1	1	-1	-1	-1	1	1	1	-1	40.78
9	1	1	-1	1	1	-1	1	-1	-1	-1	1	53.57
10	1	1	1	-1	1	1	-1	1	-1	-1	-1	74.35
11	1	-1	1	-1	-1	-1	1	1	1	-1	1	49.83
12	-1	1	-1	-1	-1	1	1	1	-1	1	1	49.57

### 3.2 Result analysis

According to the PB experiment results in Table 3, the inorganic sulfur removal rate was linearly fitted, and the relationship between the inorganic sulfur removal rate *Y* and seven factors is:

$$Y=64.2-7.07X_{A}-3.47X_{B}+5.49X_{C}-1.2X_{D}+9.87X_{E}-0.203X_{F}-4.33X_{G}$$ (7)

Table 3 listed the contribution rates of seven factors to the inorganic sulfur removal rate.

Table 4 shows that *p* < 0.01 of three factors is very significant, and the significant degree from large to small is (E) temperature (A) coal particle size, and (C) time.

**TABLE 4:** Analysis of plackett-Burman test results.

Facts	Level (-1)	Level (+1)	p	Significance
(A)Coal particle size/(mesh)	80	120+	0.001	2
(B)Bacterial solution/(ml)	10	15	0.018	5
(C)Time/(d)	7	14	0.004	3
(D)Coal consumption/(g/L)	80	120	0.225	6
(E)Temperature/(°C)	25	35	<0.001	1
(F)Shaking table speed/(r/min)	140	160	0.833	7
(G)Initial pH value	1.8	2.3	0.01	4

#### 3.2.1 Influence of temperature on inorganic sulfur removal rate

According to Arrhenius chemical reaction rate equation (Zhang et al., 2020) (Eq. 8), the higher the temperature, the faster the desulfurization reaction rate, the faster the molecular movement speed in the solution, the greater the convection diffusion speed of the solution, and the greater the inorganic sulfur removal rate.

$$k=A\cdot e^{-\frac{E}{RT}}$$ (8)

Where: *k*-reaction rate; *A*-frequency factor; *E*-apparent activation energy of reaction; *R*-ideal gas constant; *T*-temperature.

At the same time, temperature is also very important for the growth of bacteria. When the bacteria grow at the optimum temperature, the bacterial activity is the strongest, the enzymatic reaction speed in the bacteria is the fastest, and the desulfurization effect is the best. Temperature mainly affects the growth and metabolism of bacteria by influencing the kinetic parameter *K*, the maximum specific growth rate *μ*_*m*_ and the yield coefficient *Y*.

The value of *K* indicates the absorption affinity of microorganisms for nutrients. The smaller the value of *K*, the more sensitive the specific growth rate is, and the greater the absorption affinity is. *Y* represents the cell mass generated by Fe^2+^ (1 g). The greater the *Y* value, the larger the cell mass is, and the better the bacterial growth is. *μ*_*m*_ represents the maximum specific growth rate of bacteria. The larger *μ*_*m*_, the shorter cell doubling time and the faster bacterial growth.

**FIGURE 1:**
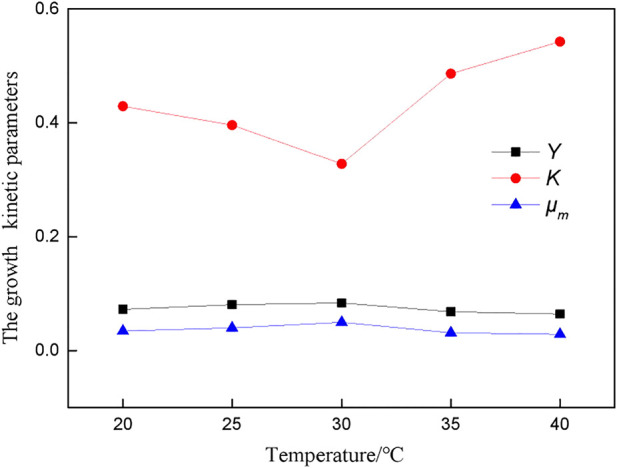
Kinetic parameters of growth at different temperatures.

The growth response model of *A.f* (Eq. 9) was used to program the growth data of bacteria at different temperatures, and the main growth kinetic parameters of bacteria were fitted, as shown in Figure 1.

$$t=\left\{\ln\frac{\rho -\left[\left(\rho_{b}^{0}/Y\right)+\rho^{0}\right]}{\rho^{0}-\left[\left(\rho_{b}^{0}/Y\right)+\rho^{0}\right]}+\frac{K}{\left(\rho_{b}^{0}/Y\right)+\rho^{0}}\times \ln\frac{\rho^{0}\left\{\rho -\left[\left(\rho_{b}^{0}/Y\right)+\rho^{0}\right]\right\}}{\rho \left\{\rho_{0}-\left[\left(\rho_{b}^{0}/Y\right)+\rho^{0}\right]\right\}}\right\}/\mu_{m}$$ (9)

Where: *ρ*_*0*_ - initial concentration of *A.f*; *ρ*_*b*_^*0*^ - initial concentration of Fe^2+^ in culture medium; *ρ* - concentration of *A.f*; *μ*_*m*_ - maximum specific growth rate; *K* - growth kinetic parameters; *Y* - yield coefficient.

Figure 1 shows that the *K* value is the smallest at 30°C, indicating that the absorption affinity of bacteria for nutrients is the strongest at this temperature. *Y* value and *μ*_*m*_ have a similar change trend. The temperature higher or lower than the optimal temperature for bacterial growth affects the growth and metabolism of bacteria, and the maximum specific growth rate and cell yield coefficient of bacteria will be reduced.

Temperature promotes the desulfurization reaction. The higher the temperature, the faster the reaction rate and the better the desulfurization effect. However, it is necessary to pay attention to the optimal temperature for bacterial growth to prevent bacterial death caused by too high temperature.

#### 3.2.2 Influence of coal particle size on inorganic sulfur removal rate

**FIGURE 2:**
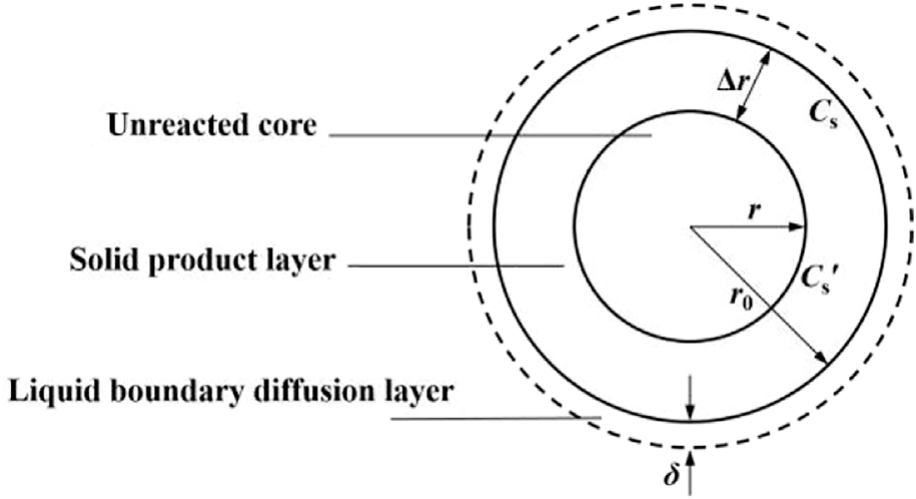
Schematic diagram of bacterial desulfurization kinetic model.

There is a liquid film layer on the surface of coal particles. When coal particles contact with solution, bacterial solution reaches the surface of coal particles for reaction through the liquid film. As the reaction progresses, reaction products are deposited on the surface of coal particles to form a solid product layer, as shown in Figure 2.

*n* is the total mole number of solid reactants in all coal particles and *A* is the total area, then the reaction rate on the coal surface can be expressed by Eq. 10.

$$\frac{dn}{dt}=-AC_{S}k_{S}$$ (10)

Where: *C*_*S*_- concentration of bacteria on coal surface; *t*-time; *k*_*S*_- Reaction rate constant for surface reactions.

There is a diffusion layer on the surface of coal particles, so the bacterial concentration in *C*_*S*_ is different from that in the main body of the solution. The diffusion of bacterial solution to the surface of coal particles can be expressed by Fick’s law (An et al., 2022).

$$J=-D\frac{C-C_{S}}{d\delta }A=\gamma \frac{dn}{dt}$$ (11)

Where: *D* - diffusion coefficient; *C*- concentration of bacteria in the main body of the solution; *δ*-thickness of boundary diffusion layer; *γ*-stoichiometric coefficient.

The diffusion rate is far less than the reaction rate, *C*_*S*_^*r*^ = 0 is visible, and the coal consumption rate is directly proportional to the diffusion amount of bacteria, which meets the reaction kinetic model of Eq. 12.

$$1-\frac{2}{3}X-{\left(1-X\right)}^{\frac{2}{3}}=\frac{2D_{2}C_{0}t}{r_{0}\alpha \rho }=k^{\prime }t$$ (12)

Where: *X*-the inorganic sulfur removal rate; *C*_*0*_- thickness of boundary diffusion layer; *r*_*0*_-initial radius of coal particles; *D*_*2*_-diffusion coefficient of bacterial solution; *α*-proportion coefficient of solid-liquid reaction consumption; *ρ*- Coal density; *t*-time; *k′*- comprehensive rate constant.

According to Eq. 12, the smaller the initial radius of coal particles, the faster the desulfurization reaction rate. Therefore, the coal particle size can directly affect the process of desulfurization reaction. At the same time, the inorganic sulfur (mainly pyrite) in coal exists in the form of inclusions with embedded particle size. When the particle size of coal reaches or is lower than the embedded particle size of pyrite, the inorganic sulfur is directly exposed on the surface of coal particles, increasing the contact area with bacteria, accelerating the reaction rate, and the inorganic sulfur removal effect is great.

#### 3.2.3 Influence of time on inorganic sulfur removal rate

**FIGURE 3:**
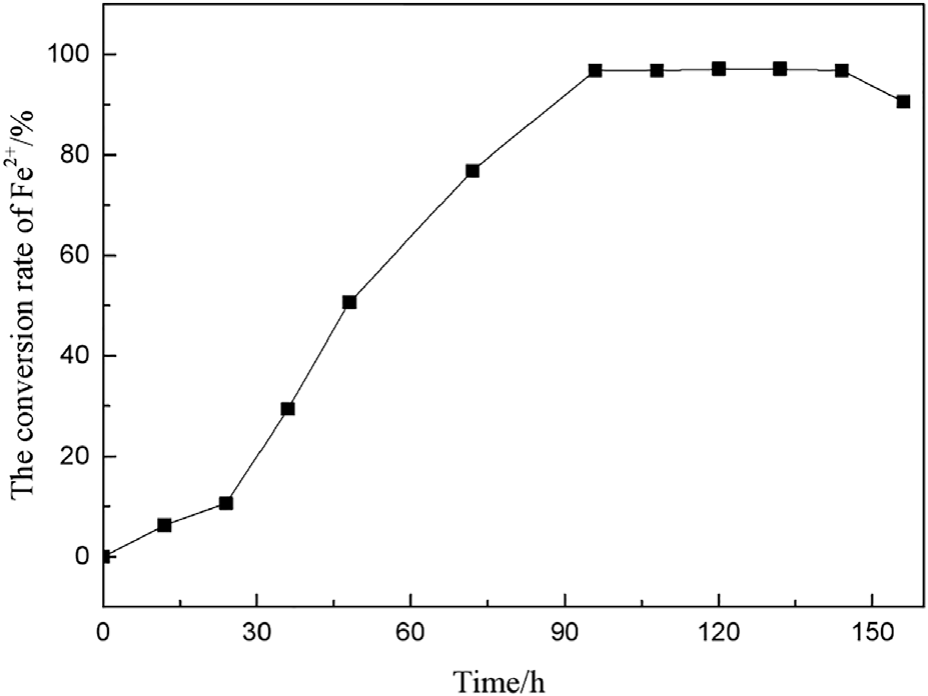
Variation trend of Fe^2+^ conversion rate over time.

*A.f* obtains energy by oxidizing Fe^2+^ to Fe^3+^, and the conversion rate of Fe^2+^ in the culture medium was measured to characterize the growth and metabolic activity of *A. f*. With sodium diphenylamine sulfonate as an indicator, the concentration of Fe^2+^ in the medium was determined by potassium dichromate titration, and the conversion trend of Fe^2+^ over time was calculated as shown in Figure 3.

Figure 3 shows that *A. f* entered the logarithmic growth period after a short adaptation period. After 96 h of culture, the Fe^2+^ conversion rate reached 95.625%. At this time, the microorganism had vigorous growth and metabolism with good activity. In the microbial desulfurization experiment, coal particles were added to the culture medium, and the microorganisms needed longer time to adapt to the growth environment.

Desulfurization time can not only affect the activity of microorganisms, but also determine the reaction degree between microorganisms and coal particles.

According to Eq. 12, the reaction rate is proportional to the time. The longer the time, the larger the area of microbe infiltrating coal particles, and the more thorough the reaction with inorganic sulfur in coal. Time not only affects the activity of microorganisms, but also determines the reaction degree between microorganisms and coal particles.

## 4 Steepest climbing experiment

The fitting of the response face equation fully restores the real situation only in the adjacent regions of the investigated regions, while the response face and the fitting equation cannot be obtained in other regions. Therefore, an effective response surface equation should be established in the regions near the maximum inorganic sulfur removal rate. In this experiment, the climbing direction were determined according to the PB experiment results, and the climbing steps were determined according to the response values of each factor.

In Eq. 7, the coefficients of factors E and C are positive and the coefficient of factor A is negative. It can be determined that the steepest climbing direction of temperature and time are positive and the steepest climbing direction of coal particle size is negative. Here, it is determined that the step size of factor E is 5, the step size of factor A is 20 and the step size of factor C is 7. The steepest climbing experiment design and results are shown in Table 5.

**TABLE 5:** Steepest climbing experiment results.

Step size	(E)Temperature/°C	(A)Coal particle size/mesh	(C)Time/d	The inorganic sulfur removal rate/%
X+1△x	25	60	7	66.42
X+2△x	30	80	14	85.38
X+3△x	35	100	21	62.80
X+4△x	45	120	28	32.88

The results of the steepest climbing experiment showed that the inorganic sulfur removal rate had the highest point between *X*+2Δ*x* and *X*+3Δ*x*. *X*+2Δ*x* was selected as the central point combined experiment center. The central point experiment conditions are as follows: temperature 30°C, coal particle size 80 mesh, time 14 days.

## 5 Response surface experiment

### 5.1 Experiment design

After the significant factors affecting the inorganic sulfur removal rate were obtained in the PB experiment, the central point of the response surface was determined by the steepest climbing experiment, and the inorganic sulfur removal rate was used as the response index for response surface experiment. Box-Behnken design (BBD) was used in the experiment, and three levels were selected for each factor, as shown in Table 6.

**TABLE 6:** Response surface factor level code.

Facts	Levels		
	-1	0	1
(x1)temperature/°C	20	30	10
(x2)coal particle size/mesh	60	80	100
(x3)time/d	7	14	21

According to the levels of the three factors, the response surface experiment scheme was obtained, as shown in Table 7.

**TABLE 7:** Response surface test design.

Serial number	Temperature/°C	Coal particle size/mesh	Time/d	The inorganic sulfur removal rate/%
1	40	60	14	43.12
2	40	80	7	45.36
3	20	80	21	63.83
4	30	80	14	76.65
5	30	80	14	80.62
6	20	100	14	68.23
7	30	100	21	87.56
8	30	60	21	73.46
9	20	80	7	60.36
10	30	80	14	78.96
11	40	100	14	53.62
12	30	100	7	74.36
13	20	60	14	63.96
14	40	80	21	55.82
15	30	60	7	75.32

The inorganic sulfur removal rate was selected as the response value in the experiment, and the relationship model between each response value *Y* and the experimental factors *x*_1_ (temperature), *x*_2_ (coal particle size), and *x*_3_ (time) was obtained by the quadratic polynomial in Eq. 13.


$$Y=\beta_{0}+\overset{n}{\underset{i=1}{\sum }}\beta_{i}x_{i}+\overset{n}{\underset{i=1}{\sum }}\beta_{ii}x_{i}^{2}+\underset{i<j}{\sum }\beta_{ij}x_{i}x_{j}$$ (13)

Where: *Y* - the predicted response value; *β*_*0*_ - coefficient constant; *β*_*i*_ - linear coefficient; *β*_*ii*_-coefficient of quadratic equation; *β*_*i j*_-interaction; *x*_*i*_、*x*_*j*_ - experiment factors code.

### 5.2 Result analysis

Response surface experiment results were shown in Table 6, and multiple quadratic regression equation was obtained by fitting, as shown in Eq. 14.

$$\begin{array}{l} Y=-58.4613+11.1520x_{1}-0.4010x_{2}-1.8869x_{3}+0.0078x_{1}x_{2}\\ +0.0250x_{1}x_{3}+0.0268x_{2}x_{3}-0.2143x_{1}^{2}-0.0002x_{2}^{2}-0.0199x_{3}^{2} \end{array}$$ (14)

**FIGURE 4:**
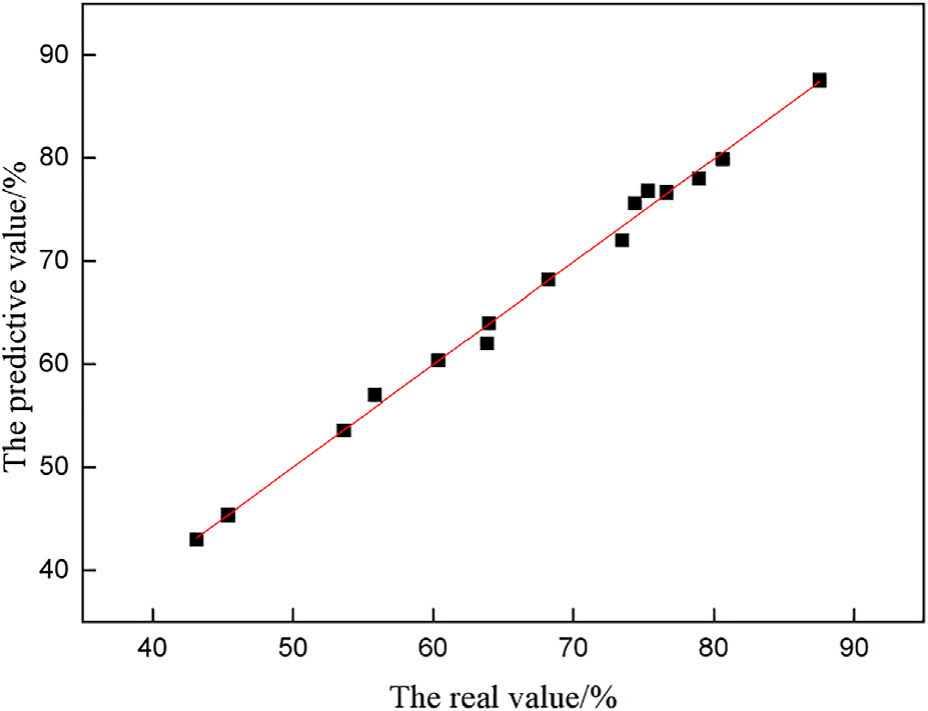
Comparison between predicted value and real value.

The fitting value of the inorganic sulfur removal rate was calculated according to Eq. 9, and the fitting value was compared with the experimental value, as shown in Figure 4.

Figure 4 shows that most coordinate points fall on or close to the line y = x, and the dispersion is small, indicating that the predicted value is close to the real value, and the fitting result is great.

Table 8 and 9 show the significance experiment and variance analysis of the regression model of the inorganic sulfur removal rate.

**TABLE 8:** Significance test of regression model.

Term	Coefficient	Coefficient standard deviation	T	p
constant	-58.4613	30.2775	-1.931	0.111
x1	11.4520	0.9201	12.120	<0.0001**
x2	-0.4010	0.5428	-0.739	0.493
x3	-1.8869	1.1153	-1.692	0.151
x12	-0.2143	0.0124	-17.257	<0.0001**
x22	-0.0002	0.0031	-0.069	0.948
x32	-0.0199	0.0253	-0.786	0.468
x1x2	0.0078	0.0060	1.306	0.249
x1x3	0.0250	0.0170	1.465	0.203
x2x3	0.0268	0.0085	3.150	0.025*

Note: superscript ** indicates that the regression analysis result is extremely significant, * indicates that the regression result is significant, and if not marked, indicates that the regression result is not significant.

**TABLE 9:** Analysis of variance of regression model.

Source	Degrees of freedom	Seq SS	Adj SS	Adj MS	F	p
Regression model	9	2387.96	2387.96	265.329	46.62	<0.0001**
Linear	3	604.37	966.47	322.156	35.39	<0.001**
Square	3	1705.19	1705.19	568.398	99.86	<0.0001**
Interaction	3	78.39	78.39	26.131	4.59	0.067
Mismatch term	3	20.51	20.51	6.836	1.72	0.388
Residual	5	28.46	28.46	5.692		
Pure error	2	7.95	7.95	3.975		
Total	14	2416.42				

Note: superscript ** indicates that the regression analysis result is extremely significant, * indicates that the regression result is significant, and if not marked, indicates that the regression result is not significant.

Table 8 shows that the *p* of factors *x*_*1*_, *x*_*1*_^*2*^ and *x*_*2*_ *x*_*3*_ are less than 0.05, which are significant factors. Table 9 shows that the regression model *p* < 0.0001 and the mismatch term *p* is 0.388 > 0.05, indicating that the regression of the model is extremely significant and the mismatch is not significant, this experimental method is reliable. The model correlation coefficient *R* ^*2*^ = 96.45%, indicating that the correlation is very great.

### 5.3 Response surface analysis

**FIGURE 5:**
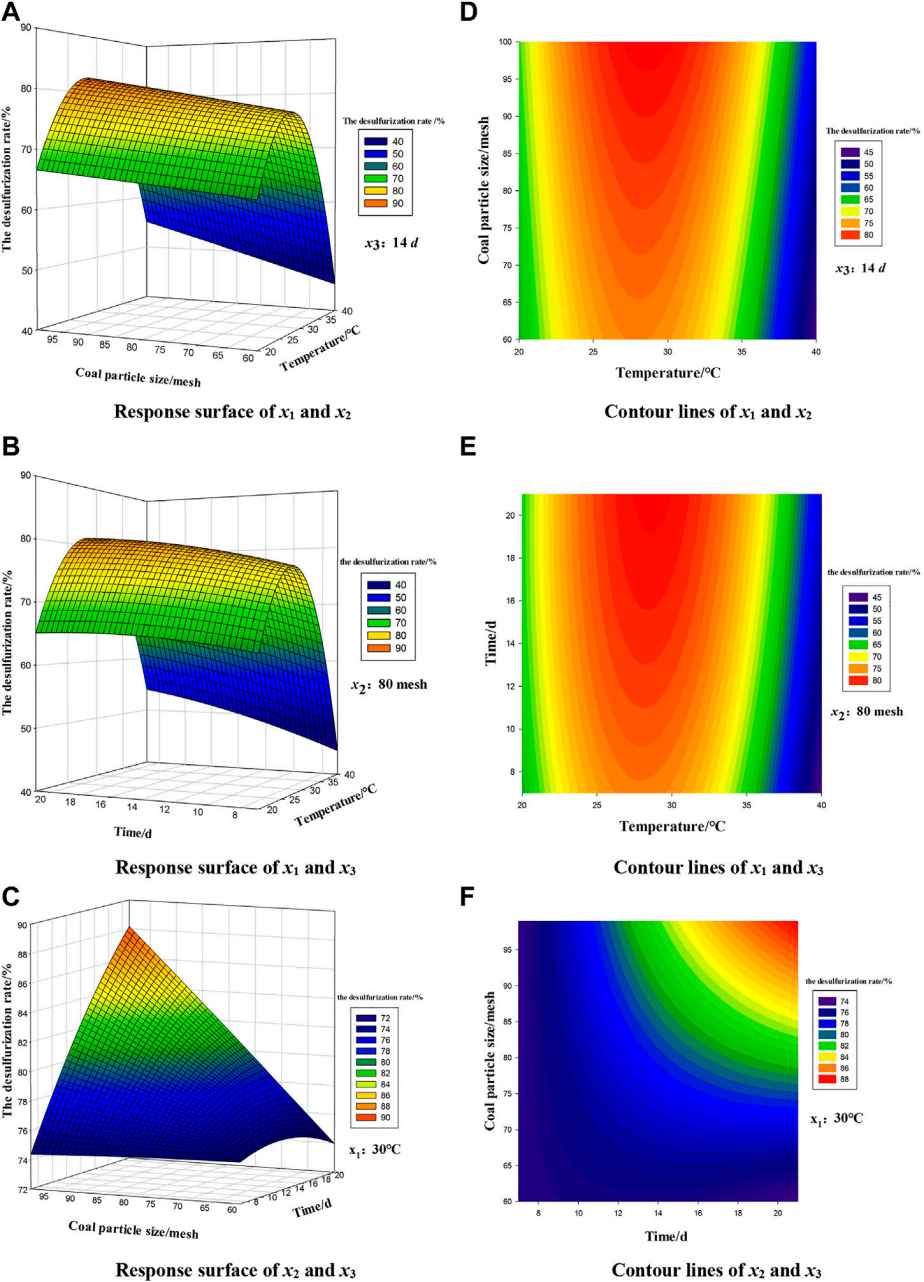
Contour lines and response surfaces between variables.

Figure 5 shows the response surface and contour map of the interaction of experiment factors *x*_1_ (temperature), *x*_2_ (coal particle size) and *x*_3_ (time). This graph can not only predict and optimize the response value, but also analyze the interaction of any two factors to obtain the interaction rule.

Figure 5 shows that the change trend of contour lines and response surface between various factors indicates the strength of interaction. The smoothness of the surface can reflect the significance and size of the interaction, the greater the curvature of the surface, the greater the interaction. According to Figure 5D–F, *x*_1_ (temperature) and *x*_2_ (coal particle size) have the most obvious interaction among the three contour lines. By response surface between the variables, (Figures 5A–C), the surface A is the steepest, the surface B is the second, and the surface C is the most flat, therefore the interaction between *x*_1_ (temperature) and *x*_2_ (coal particle size) has the greatest impact on the inorganic sulfur removal rate, followed by the interaction between *x*_1_ (temperature) and the *x*_3_ (time), and the weakest is *x*_2_ (coal particle size) and *x*_3_ (time). Therefore, *x*_1_ (temperature) fundamentally determines the inorganic sulfur removal rate.

### 5.4 Optimization experiment verification

Nonlinear programming with constraints was carried out by limiting constraints. The optimization scheme and results are shown in Table 10.

**TABLE 10:** Optimal parameters design of the inorganic sulfur removal rate.

Parameter	Temperature/°C	Coal particle size/mesh	Time/d	The inorganic sulfur removal rate/%	
				Predicted value	Real value
Maximum value	40	100	7		
Minimum value	20	60	21		
Optimization scheme	29.50	100	11.67	79.99	79.78

Table 10 shows that the optimal temperature is 29.50°C, the optimal coal particle size is 100 mesh, and the optimal time is 11.67 days. Under this condition, the predicted value of the inorganic sulfur removal rate is 79.99%, and the error with the real value is only 0.21%, indicating that the model is relatively reliable and can well predict the inorganic sulfur removal rate under different conditions.

## 6 Conclusion

1) The Plackett-Bruman experiment shows that the three most significant factors affecting the inorganic sulfur removal rate in coal are temperature, coal particle size and time. The center point of the three factor response surface experiment is obtained through the steepest climbing experiment: the temperature is 30°C, the coal particle size is 80 mesh, and the time is 14 days.
2) Through the analysis of variance, significance experiment, contour lines and response surface diagram, it is known that temperature is the fundamental factor determining the inorganic sulfur removal rate in coal.
3) Using response surface design optimization, the optimum test conditions are put forward: the temperature is 29.50°C, the coal particle size is 100 mesh, the desulfurization time is 11.67 days, the amount of bacterial solution is 15 ml, the amount of coal is 80 g, the rotating speed of shaking table is 140 r/min, and the initial pH value is 2.0. Under this condition, the removal rate of inorganic sulfur in coal reaches 79.78%, which is consistent with the predicted value.
4) Response surface design optimization is used to propose the optimal experiment conditions: temperature is 29.50°C, coal particle size is 100 mesh, time is 11.67 days, bacterial solution is 15 ml, coal consumption is 80 g, shaking speed is 140 r/min, initial pH value is 2.0. Under these conditions, the inorganic sulfur removal rate in coal reaches 79.78%, which is consistent with the predicted value.

## Data Availability

The raw data supporting the conclusions of this article will be made available by the authors, without undue reservation.
